# Rumination mediates the relationship between overgeneral autobiographical memory and depression in patients with major depressive disorder

**DOI:** 10.1186/s12888-017-1264-8

**Published:** 2017-03-21

**Authors:** Yansong Liu, Xinnian Yu, Bixiu Yang, Fuquan Zhang, Wenhua Zou, Aiguo Na, Xudong Zhao, Guangzhong Yin

**Affiliations:** 1 Department of Psychosomatic Medicine, Shanghai East Hospital, Tongji University School of Medicine, Shanghai, 200092 China; 2 Wuxi Institute of Technology, Wuxi, 214121 Jiangsu Province China; 3 Wuxi Mental Health Center, Wuxi, 214151 Jiangsu Province China; 4 Students Affairs Office, Suzhou Institute of Instruction and Communications, Suzhou, 215000 Jiangsu Province China; 5 Department of Psychiatry, Mental Hospital of Jiangsu Province Prison Administration, Nanjing, 210000 Jiangsu Province China; 6 Suzhou Guangji Hospital, Suzhou, 215008 Jiangsu Province China

**Keywords:** Overgeneral autobiographical memory, Rumination, Depressive symptoms, Mediation analysis

## Abstract

**Background:**

Overgeneral autobiographical memory has been identified as a risk factor for the onset and maintenance of depression. However, little is known about the underlying mechanisms that might explain overgeneral autobiographical memory phenomenon in depression. The purpose of this study was to test the mediation effects of rumination on the relationship between overgeneral autobiographical memory and depressive symptoms. Specifically, the mediation effects of brooding and reflection subtypes of rumination were examined in patients with major depressive disorder.

**Methods:**

Eighty-seven patients with major depressive disorder completed the 17-item Hamilton Depression Rating Scale, Ruminative Response Scale, and Autobiographical Memory Test. Bootstrap mediation analysis for simple and multiple mediation models through the PROCESS macro was applied.

**Results:**

Simple mediation analysis showed that rumination significantly mediated the relationship between overgeneral autobiographical memory and depression symptoms. Multiple mediation analyses showed that brooding, but not reflection, significantly mediated the relationship between overgeneral autobiographical memory and depression symptoms.

**Conclusions:**

Our results indicate that global rumination partly mediates the relationship between overgeneral autobiographical memory and depressive symptoms in patients with major depressive disorder. Furthermore, the present results suggest that the mediating role of rumination in the relationship between overgeneral autobiographical memory and depression is mainly due to the maladaptive brooding subtype of rumination.

## Background

Autobiographical memory (AM) is a uniquely human form of memory that includes both specific episodic memories of past events and more conceptual, self-relevant information [1–3]. A specific AM is a memory for an event that happened at a particular time and place and lasted for a day or less (e.g., “I played football with my colleagues last Saturday afternoon”). In contrast, an overgeneral autobiographical memory (OGM) consists of categoric memories that refer to a class of repeated events (e.g., “I used to play volleyball every Thursday”) and extended memories that refer to events that lasted longer than a day (e.g., “When I was on a cruise this winter vacation”).

Over the past 20 years, a large number of studies have shown OGM is a robust and replicable phenomenon in patients with major depressive disorder (MDD) and can predict the course of depression [2, 4, 5]. Moreover, studies have shown that OGM predicts the level of depressive symptoms at follow-up in clinical samples of patients with MDD [6, 7] and in nonclinical samples of adolescents [8, 9]. In addition, Hermans et al. [10] found that patients with MDD who exhibited higher levels of OGM upon hospital admission were more likely than patients not characterized by OGM at the initial assessment to still meet criteria for MDD 3–4 weeks later. Taken together, these studies suggest that OGM plays a strong role in the onset and maintenance of depression. Although OGM has been regarded as a risk factor for the onset and maintenance of depression, little is known about the underlying mechanisms that might explain OGM phenomenon in depression. Recent studies suggest that rumination might be a possible mechanism that explains how OGM leads to depression [11, 12].

Rumination can be defined as repetitive and passive thoughts that focus one’s attention on one’s depressive symptoms as well as the possible causes and consequences of those symptoms [13]. It has been found to be associated with more severe depressive symptoms and a heightened vulnerability to experience major depressive episodes [14, 15]. Previous studies have shown that OGM and rumination are significantly related and mutually reinforced. Raes et al. [16] found that rumination was associated with OGM in patients with MDD. Watkins et al. [17] found that reducing rumination, either using distraction or self-focus manipulation, reduced OGM in depressed participants. These studies suggest rumination leads to OGM. However, on the other hand, several researchers found that OGM also contributed to rumination. Raes et al. [18] were the first to show that OGM causally influenced rumination by experimental manipulation. Furthermore, Raes et al. [12] found rumination mediated the relationship between OGM and depression at a 7-month follow-up in a sample of 28 patients with MDD. However, the sample size of the study was relatively small. Therefore, it seems essential to replicate this finding in a larger group of patients with MDD. The first aim of this study was to replicate this clinical finding in a large sample of patients with MDD. We hypothesized that global rumination would mediate the relationship between OGM and depressive symptoms.

Rumination has been dismantled into two distinct subtypes: brooding, which represents a tendency toward mood pondering (e.g., “why do I have problems other people don’t have?”), and reflection, which indicates a tendency to contemplate and reflect (e.g., “analyse your personality to try to understand why you are depressed”) [19]. Several studies have found that brooding and reflection can be considered as, respectively, a maladaptive and an adaptive component of rumination [20, 21]. With regard to the relationship between OGM and rumination components, Debeer et al. [22] found that brooding, but not reflection, was significantly associated with OGM and functioned as a mediator between reduced OGM and depression in a non-clinical sample. In a sample of dysphoric participants, Romero et al. [23] also found that brooding was significantly associated with OGM, whereas reflection was not significantly associated with OGM.

However, no studies have yet explored the mediation effects of brooding and reflection on the relationship between OGM and depressive symptoms in a sample of patients with MDD. To the best of our knowledge, the present study is the first to fill this gap in the literature by trying to replicate this clinical finding in patients with MDD. The second aim of this study was to investigate whether OGM holds different relationships with the brooding and reflection subtypes of rumination in patients with MDD. Based on the above literature review, we hypothesized that brooding, and not reflection, would mediate the relationship between OGM and depressive symptoms.

## Methods

### Participants

Eighty-seven patients with MDD (56 females and 31 males) were recruited from three psychiatric hospitals in Jiangsu Province. All the patients were diagnosed based on the Structured Clinical Interview for DSM-IV (SCID-I) by experienced psychiatrists. Inclusion criteria were as follows: (1) Diagnostic and Statistical Manual of Mental Disorders, Fourth Edition (DSM-IV) criteria for MDD; (2) at least a total score of 17 on the 17-item Hamilton Depression Rating Scale (HDRS); (3) age between 18 and 65 years. Exclusion criteria were as follows: (1) had substantial head injury or neurological disorder; (2) had substance abuse or dependence; (3) had other psychiatric disorders, such as schizophrenia or bipolar disorder; (4) received electroconvulsive therapy in the 6 months prior to the study. The kappa value for inter-rater reliability of MDD using this method was 1.00.

The age of participants ranged from 18 to 65 years [mean (M) = 40.92, standard deviation (SD) = 12.67]. The educational level of participants ranged from 6 to 19 years (M = 11.94, SD = 3.40). Illness durations ranged from 1 to 19 months (M = 7.16, SD = 3.70). In addition, it is worth mentioning that with 87 participants, the present study had at least 0.80 power to detect a medium effect size [24].

This study was approved by the Ethics Committees of the Wuxi Mental Health Center, and signed informed consent was obtained from all participants. All study procedures were in accordance with the Declaration of Helsinki.

### Measures

#### Ruminative Response Scale (RRS)

The Ruminative Response Scale (RRS) was used to assess ruminative responses to depressed mood. The RRS is composed of 22 items that are self-focused, symptom focused, or focused on possible consequences and causes of mood. Responses are scored on a four-point Likert scale ranging from 1 (“almost never”) to 4 (“almost always”). The Brooding and Reflection subscales of the RRS each contain five items. The RRS, Brooding and Reflection subscales have shown good test-retest reliability and internal consistency [13, 19].

#### 17-item Hamilton Depression Rating Scale

The 17-item Hamilton Depression Rating Scale (HDRS) is a test measuring the severity of depressive symptoms. It is widely used in research on mood disorders and in clinical practice. The HDRS has been found to have good validity (correlation coefficient for HDRS and clinical changes = 0.26) and reliability (*r* = 0.88–0.99) in Chinese populations [25].

#### Autobiographical memory test

Following the Chinese version of the Autobiographical Memory Test (AMT) [26, 27], participants were asked to retrieve a specific memory to 12 given emotional cue words in Chinese. Cue words were presented orally in a fixed order, with six negative and six positive words alternating: guilty, successful, lonely, honest, sad, proud, horrible, brave, angry, interested, painful, and happy. Negative and positive cue words were matched for word imageability, emotionality, and frequency. Participants were given 60 s to recall a specific memory for each cue. If participants did not recall a specific memory, they were verbally prompted to describe a particular place or a particular time. The prompting procedure was repeated until the participant retrieved a specific memory or until the time limit was exceeded. Before testing, participants were given two practice words (relaxed, ugly) to familiarize them with the procedure.

Each response was later coded as either a specific memory or an OGM; the latter were further qualified as a categorical memory (e.g., “parties with my classmates”), an extended memory (e.g., “when I was on winter vacation last month”), or no memory (e.g., “semantic information and omission”). The “OGM” measure used in all the analyses was the number of responses falling into the repeated, extended and no memory categories. Given that OGM has been found to be a robust and replicable phenomenon in patients with MDD [5, 28], we used the number of OGM as the outcome measure. Using this scoring procedure, we obtained good reliability (*K* = 0.91).

### Statistical analysis

Correlation analysis of the five variables (i.e. HDRS, OGM, RRS, brooding, reflection) was conducted using SPSS 15.0 software (SPSS Inc., Chicago, IL, USA). A *P* value less than 0.05 was considered statistically significant. Bonferroni correction was used for multiple comparisons. To examine whether rumination, and more specifically its brooding and reflection components, would mediate the relationship between OGM and depressive symptoms in patients with MDD, bootstrap mediation analysis for simple and multiple mediation through the SPSS PROCESS macro was applied [29]. Bootstrap mediation, a nonparametric sampling procedure, does not impose the assumption of normality of the sampling distribution of the indirect effect, and therefore is considered to be more powerful for hypothesis testing for mediation analysis than the Sobel’s test, which assumes a normal distribution of the indirect effect [29, 30]. Moreover, the SPSS PROCESS macro applied in the present study allows for the testing of multiple mediators simultaneously (in the present study, both brooding and reflection). We utilized 5000 bootstrap samples for coefficient and indirect estimation. The indirect effect was statistically significant if the 95% bias-corrected confidence intervals (CIs) for the indirect effect did not include zero [29, 30]. The completely standardized effect size (CS) and kappa-squared (K^2^) will be reported as indices of effect size [31].

Simple and multiple mediation models were constructed to test our hypotheses. Simple mediation analysis was used to examine whether global rumination would mediate the relationship between OGM and depressive symptoms. Multiple mediation analyses were used to test whether brooding and reflection mediated the effects of OGM on depressive symptoms. In addition, we tested reverse mediation models with OGM entered as a proposed mediator of the relationship between rumination and depressive symptoms. We also planned reverse mediation models with rumination entered as a mediator of the relationship between depressive symptoms and OGM. Considering that age and education level were correlated with OGM [32], both of these variables were entered as covariates in all mediation analyses.

## Results

### Correlations

The Pearson correlations, mean scores and SDs for each of the five variables (i.e. HDRS, OGM, RRS, brooding, reflection) are presented in Table 1. RRS was positively correlated with OGM (*r* = 0.65, *p <* 0.001) and HDRS (*r* = 0.53, *p <* 0.001). Brooding was positively correlated with OGM (*r* = 0.59, *p <* 0.001) and HDRS (*r* = 0.54, *p <* 0.001). Reflection was positively correlated with OGM (*r* = 0.34, *p <* 0.01) and HDRS (*r* = 0.31, *p <* 0.01). After Bonferroni correction for multiple comparisons (*P <* 0.05/10 = 0.005), RRS and brooding were also significantly positively correlated with OGM and HDRS, whereas reflection was not significantly correlated with OGM and HDRS.

**Table 1:** Means, standard deviations (SDs) and correlations of the measured variables (*N* = 87)

Variable	Mean	*SD*	1	2	3	4	5
1. HDRS	25.82	5.15	1	0.56^**^	0.53^**^	0.54^**^	0.31^*^
2. OGM	5.33	2.47		1	0.65^**^	0.59^**^	0.34^*^
3. RRS	51.33	11.88			1	0.68^**^	0.52^**^
4. Brooding	11.65	3.77				1	0.19
5. Reflection	10.53	3.17					1

*Note. HDRS* 17-item Hamilton Depression Rating Scale, *OGM* Overgeneral Autobiographical Memory, *RRS* Ruminative Response Scale, *Brooding* Brooding Subscale of Ruminative Response Scale, *Reflection* Reflection Subscale of Ruminative Response Scale

^*^ *p* < 0.01. ^**^ *p* < 0.001

Correlations at ^*^ *p* < 0.01 did not survive the Bonferroni correction for multiple comparisons

### Simple mediation effects of rumination on the relationship between OGM and depressive symptoms

**Fig. 1:**
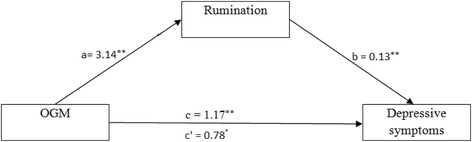
Simple mediation model for OGM and depressive symptoms via rumination. Unstandardized path coefficients indicated above. c = total effect; c´ = direct effect. ^*^ *P* < 0.01; ^**^ *P* < 0.001

As expected, simple mediation analysis showed that OGM had a significant effect on depressive symptoms via rumination (point estimate = 0.39, 95% CI [0.07, 0.77]). The effect sizes for the indirect effect (CS = 0.19, K^2^ = 0.17) suggest a medium effect size according to Cohen’s conventions [33]. Higher levels of OGM were associated with higher levels of rumination (a = 3.14, standard error (SE) = 0.40, *p <* 0.001, 95% CI [2.35, 3.93]). Higher levels of rumination were also associated with higher levels of depressive symptoms (b = 0.13, SE = 0.05, *p <* 0.001, 95% CI [0.03, 0.22]). OGM had a significant positive total effect on depressive symptoms (c = 1.17, SE = 0.19, *p <* 0.001, 95% CI [0.80, 1.54]). With the addition of rumination to the model, the direct effect of OGM on depressive symptoms was significant (c´ = 0.78, SE = 0.24, *p <* 0.01, 95% CI [0.30, 1.25]). The results supported the notion that global rumination partly mediated the relationship between OGM and depressive symptoms in patients with MDD. Figure 1 shows the results of the simple mediation analysis examining rumination as a mediator in the relationship between OGM and depressive symptoms.

### Multiple mediation effects of brooding and reflection on the relationship between OGM and depressive symptoms

**Fig. 2:**
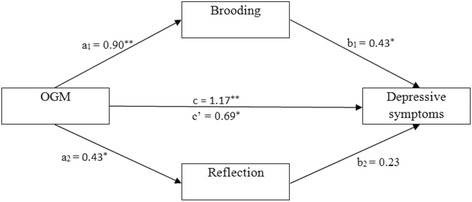
Multiple mediation model for OGM and depressive symptoms via brooding and reflection. Unstandardized path coefficients indicated above. c = total effect; c´ = direct effect. ^*^ *P* < 0.01; ^**^ *P* < 0.001

Multiple mediation analyses showed that the total indirect effect of OGM on depressive symptoms through brooding and reflection was significant (point estimate = 0.48, 95% CI [0.23, 0.86]). The effect sizes for the total indirect effect (CS = 0.23) suggest a medium effect size according to Cohen’s guidelines [33]. OGM was positively associated with brooding (a_1_ = 0.90, SE = 0.13, *p <* 0.001, 95% CI [0.63, 1.17]) and positively associated with reflection (a_2_ = 0.43, SE = 0.13, *p <* 0.01, 95% CI [0.17, 0.69]). Brooding positively predicted depressive symptoms (b_1_ = 0.43, SE = 0.14, *p <* 0.01, 95% CI [0.14, 0.71]), and the path from reflection to depressive symptoms was not significant (b_2_ = 0.23, SE = 0.15, *p <* 0.05, 95% CI [-0.07, 0.52]). OGM had a significant positive total effect on depressive symptoms (c = 1.17, SE = 0.19, *p <* 0.001, 95% CI [0.80, 1.54]). With the addition of brooding and reflection to the model, the direct effect of OGM on depressive symptoms was also significant (c´ = 0.69, SE = 0.23, *p <* 0.01, 95% CI [0.23, 1.14]). The specific indirect effect of OGM on depressive symptoms through brooding was also significant (point estimate = 0.38, 95% CI [0.13, 0.73]), whereas the specific indirect effect of OGM on depressive symptoms via reflection was not significant (point estimate = 0.10, 95% CI [-0.01, 0.30]). The findings indicated that brooding only partially mediated the relationship between OGM and depressive symptoms. Figure 2 displays results of the multiple mediation analyses examining brooding and reflection simultaneously as mediators of the relationship between OGM and depression symptoms.

### Reverse mediation analyses

To explore the directional specificity of these effects, we ran reverse mediation models examining OGM as a mediator of the relationship between rumination and depressive symptoms. The results showed that OGM did not mediate the relationship between rumination and depressive symptoms (point estimate = 0.17, 95% CI [-0.06, 0.48]), and the direct effect of rumination on depressive symptoms was significant (c´ = 0.71, SE = 0.23, *p <* 0.01, 95% CI [0.24, 1.18]). Moreover, we also ran similar reverse mediation models to investigate whether rumination mediated the relationship between depressive symptoms and OGM. Rumination did not show a significant indirect effect on the relationship between depressive symptoms and OGM (point estimate = 0.29, 95% CI [-0.08, 0.89]). The direct effect of depressive symptoms on OGM was significant (c´ = 2.15, SE = 0.49, *p <* 0.01, 95% CI [1.18, 3.12]). These findings support the mediation effects of rumination on the relationship between OGM and depressive symptoms in patients with MDD.

## Discussion

The purpose of this study was to test the mediation effects of rumination on the relationship between OGM and depressive symptoms. Specifically, the mediation effects of brooding and reflection subtypes of rumination were examined in patients with MDD.

As expected, OGM showed a significant correlation with global rumination. Global rumination was found to partly mediate the relationship between OGM and depressive symptoms in patients with MDD. This finding is in line with Williams’s [2, 34] hypothesis that both OGM and rumination are closely related and mutually reinforced. On the one hand, rumination contributes to OGM. On the other hand, OGM also leads to rumination. Raes et al. [18] found that induction of an OGM retrieval style, compared to a specific retrieval style, increases the extent to which ruminators’ mental mode is in line with rumination in a sample of students. Moreover, previous studies have shown that global rumination mediates the relationship between OGM and depressive symptoms in a clinical sample of patients with MDD [12] and in a nonclinical sample of students [22]. In addition, since the reverse relationships (OGM mediating the relationship between rumination and depressive symptoms and rumination mediating the relationship between depressive symptoms and OGM) were not significant, these results also support the mediation effects of rumination on the relationship between OGM and depressive symptoms in patients with MDD. Thus, the findings suggest individuals with higher levels of OGM tended to engage in rumination, which in turn predicted more depressive symptoms.

As hypothesized, OGM showed a significant correlation with brooding. Mediation analyses revealed that brooding was significantly associated with OGM and functioned as a mediator between OGM and depression in patients with MDD. The finding is consistent with previous studies that found brooding was significantly associated with OGM in nonclinical participants [22, 23]. Moreover, brooding is considered as a maladaptive component of rumination and accounts for the deleterious outcomes in depression. In addition, previous studies have found that the maladaptive brooding rumination in patients with MDD leads to OGM because it reduces working memory functioning [2, 23]. In addition, our results indicated that reflection was not significantly correlated with OGM and depression. This result is consistent with previous studies that found reflection was not significantly related to memory performance in nonclinical participants [22, 23]. As a matter of fact, reflection is defined as an adaptive component of rumination and can alleviate one’s depressive symptoms [11, 19]. This result supports this perspective.

The present findings indicate that the mediator role of rumination in the relationship between OGM and depression is mainly due to its maladaptive brooding component. The present findings also show that individuals who exhibited higher levels of OGM tended to engage in brooding, and in turn, brooding enhanced their depressive symptoms.

There are several limitations to the present study that we need to address. First, because this study was a cross-sectional design, it did not allow us to infer causality. Specifically, the mediators may just be covariates rather than mediators [35]. Therefore, future studies should employ a longitudinal design to investigate the effect of rumination on the relationship between OGM and depression. Second, we lacked a healthy control group. However, previous studies have shown that rumination and the brooding subtype of rumination mediated the relationship between OGM and depressive symptoms in a non-clinical sample [22]. Third, only rumination and its two subtypes were investigated as potential mediators of the relationship between OGM and depressive symptoms. Future studies should examine additional factors (e.g., negative cognitive styles and stress factors). Finally, the study was conducted in a clinical sample of patients with MDD, which consequently limits the generalizability of the present findings to the healthy population. Future studies should examine to what extent the relationships reported in this study generalize to a sample more representative of the general population.

## Conclusion

In conclusion, our results indicate that global rumination partly mediates the relationship between OGM and depressive symptoms in patients with MDD. Furthermore, the present results suggest that brooding, but not reflection, partly mediates the relationship between OGM and depressive symptoms.

The present findings have important clinical implications for understanding how OGM leads to depressive symptoms in patients with MDD. Based on the current findings, we can develop targeted interventions and improve efficacy of existing interventions. Moreover, as implied by the effects of reflection, interventions should encourage patients to focus on their problems purposefully in the treatment process.

## Abbreviations

AMT: Autobiographical memory test
CI: Confidence interval
HDRS: 17-item Hamilton Depression Rating Scale
M: Mean
MDD: Major depressive disorder
OGM: Overgeneral Autobiographical Memory
RRS: Ruminative Response Scale
SD: Standard deviation
SE: Standard error
